# Guanosine-Based Nucleotides, the Sons of a Lesser God in the Purinergic Signal Scenario of Excitable Tissues

**DOI:** 10.3390/ijms21051591

**Published:** 2020-02-26

**Authors:** Rosa Mancinelli, Giorgio Fanò-Illic, Tiziana Pietrangelo, Stefania Fulle

**Affiliations:** 1 Department of Neuroscience Imaging and Clinical Sciences, University “G. d’Annunzio” of Chieti-Pescara, 66100 Chieti, Italy; rosa.mancinelli@unich.it (R.M.); tiziana.pietrangelo@unich.it (T.P.); 2 Interuniversity Institute of Miology (IIM), 66100 Chieti, Italy; fanoillic@gmail.com; 3 Libera Università di Alcatraz, Santa Cristina di Gubbio, 06024 Gubbio, Italy

**Keywords:** guanine-based purines (GBPs), purinergic signaling, purine receptors

## Abstract

Purines are nitrogen compounds consisting mainly of a nitrogen base of adenine (ABP) or guanine (GBP) and their derivatives: nucleosides (nitrogen bases plus ribose) and nucleotides (nitrogen bases plus ribose and phosphate). These compounds are very common in nature, especially in a phosphorylated form. There is increasing evidence that purines are involved in the development of different organs such as the heart, skeletal muscle and brain. When brain development is complete, some purinergic mechanisms may be silenced, but may be reactivated in the adult brain/muscle, suggesting a role for purines in regeneration and self-repair. Thus, it is possible that guanosine-5′-triphosphate (GTP) also acts as regulator during the adult phase. However, regarding GBP, no specific receptor has been cloned for GTP or its metabolites, although specific binding sites with distinct GTP affinity characteristics have been found in both muscle and neural cell lines. Finally, even if the cross regulation mechanisms between the two different purines (ABP and GBP) are still largely unknown, it is now possible to hypothesize the existence of specific signal paths for guanosine-based nucleotides that are capable of modulating the intensity and duration of the intracellular signal, particularly in excitable tissues such as brain and muscle.

## 1. Overview

Purines are endogenous organic molecules that are essential for all cells. Purines, adenine-based purines (ABPs) and guanine-based purines (GBPs), are composed of two fused linked rings containing five carbon and four nitrogen atoms as well as their derivatives: nucleosides (nitrogenous bases plus a pentose sugar, commonly ribose); and nucleotides (nitrogenous bases plus ribose and phosphate) that are mono-, di- or tri-phosphorylated [1]. Adenine and guanine are very commonly found in nature, mostly as nucleoside and nucleotide derivatives, i.e., phosphorylates (Adenosine monophosphate, AMP; Adenosine diphosphate ADP; Adenosine triphosphate, ATP; Guanosine monophosphate, GMP; Guanosine diphosphate, GDP; Guanosine triphosphate, GTP). Both nucleosides and nucleotides are multifunctional molecules involved in numerous cellular processes, mainly as building blocks of nucleic acid but also as important regulators of different biochemical pathways acting as energy suppliers and/or allosteric modulators of enzymatic activity.

**Figure 1:**
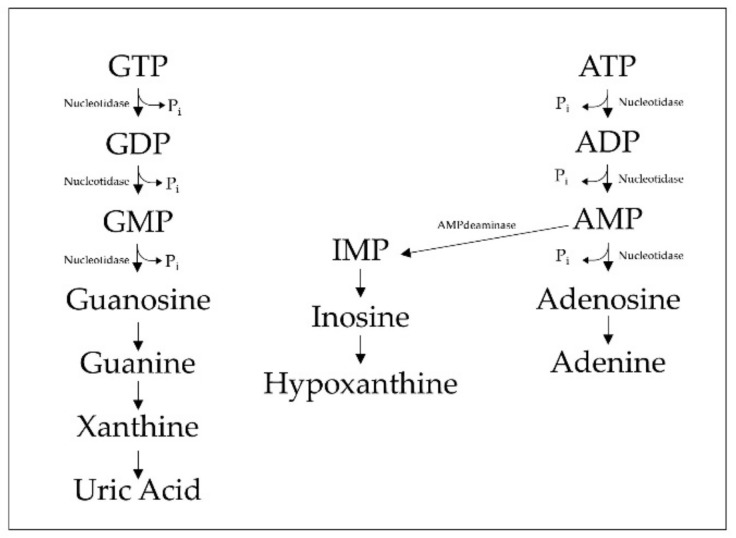
Purine metabolism. The guanine nucleotides guanosine-5′-triphosphate (GTP), guanosine-5′-diphosphate (GDP) and guanosine-5′-monophosphate (GMP) are sequentially dephosphorylated by ecto-nucleotidases thus generating guanosine. Further enzymatic reactions convert Guanosine into guanine, which is then converted into xanthine and subsequently to uric acid. Adenine nucleotides are also hydrolyzed, forming the nucleosides adenosine and inosine. In the complex scenario of cell-to-cell signaling, the first evidence of a role for nucleotides, mainly Adenosine triphosphate (ATP) and nucleoside, was provided by Geoffrey Burnstock and collaborators who first created the term “purinergic signaling” in the 1970s [3]. After an initial reluctance about the unreliable possibility that ATP was released into the extracellular environment from undamaged cells, the hypothesis that ATP could serve as an extracellular messenger gained strength, until it became what it is today: the solution to many signaling problems of which we do not have sufficient knowledge. The final confirmation of the purinergic hypothesis was provided by the cloning of the ATP receptors, initially the metabotropic ones (P2Y) and then the ionotropic (P2X). Today, at least fifteen P2 receptors for nucleotides and four P1 receptors for nucleosides with different functions (from neurotransmission in central and neuromuscular synapses to endocrine secretion, vasodilation, immune response, etc.) and widespread distribution in different organs and tissues have been identified [4,5].

To maintain the balance of purine nucleotides, cells have developed systems of interconversion between them, which always lead to the production of inosine-5-monophosphate (IMP); indeed, there is no way to convert AMP to GMP with a single reaction or vice versa. Adenyl nucleotides are transformed both by nucleotidase into adenosine, with the release of inorganic phosphate, and by AMP deaminase (specifically using AMP) into IMP, with the release of an amino group. Guanyl nucleotides are transformed by nucleotidase into guanosine with release of inorganic phosphate (Figure 1) [2].

Extracellularly, adenosine concentration could be considered as a homeostatic value while considering blood compartments. Most of the extracellular adenosine derives from the release and metabolism of adenine nucleotides. Nucleotides are generally released by damaged and dead cells and in a regulated manner by exocytosis, in particular by exocytotic granules, membrane-derived microvesicles, membrane channels and specific ATP transporters, as well as by exosomes [5]. Then, firstly the extracellular pool of adenosine is reduced mainly through nucleoside transporters that transport it back into the cell; secondly, adenosine is deaminated to inosine through the action of adenosine deaminase. Finally, adenosine could be deactivated by its phosphorylation in AMP through the action of adenosine kinase [6]. Adenosine acts via specific receptors that are divided into at least four categories: A(1), A(2A), A(2B) and A(3) [7]. The activation of the mechanisms of generation and elimination of adenosine leads to an extracellular concentration of purine in the range 10–200 nM, which can rise to 10–100 μM under stressful conditions [8].

In order to simplify a sometimes even contradictory receptor framework, in 2009 Volontè and D’Ambrosi introduced the concept of purinoma with which the ligand-receptor system is not seen as a single means of communication but rather as a terminal of a complex organization.

This system presents many aspects that correlate with each other. This organization could explain heterogeneous, and even opposing, cellular responses, which instead should appropriately refer to epigenetic factors and/or to changes in the extracellular environment (niche) [9].

Thus, the system becomes the terminal of a molecular network that connects the first messengers (hormones or growth factors) to their specific receptors, converging them to a single intracellular effector mechanism. According to the proponents, therefore, ‘purinoma’ is the molecular complex responsible for the ‘converging’ biological effects of the extracellular purine and pyrimidine ligands. In addition to a wide range of purinergic ligands, purinoma consists of ectonucleotide-metabolizing enzymes that hydrolyze nucleoside phosphates, purinergic receptors classified as P1 and P2, nucleoside trans membrane transporters and nucleotide channels and transporters.

In recent years, numerous studies have shown that nucleotides can act as modulators of the release of intercellular messengers in the surrounding environment of the operating cell. Realistically, the understanding of the purinergic transductive mechanism is complicated due to several factors: cells can release different nucleotides, express different subtypes of the same receptor with different specificity and/or affinity for the ligand. In this scenario, the intracellular responses can be variegated. Taking this into account, one of the main role of the extracellular nucleotides could be controlling a cascade of multiple set of cellular functional mechanisms [10].

The activation of the system does not independently occur but through closely concerted actions that under physiological conditions, leads to a unique effect. This means that the system is organized in such a way that it is not intended to be composed of separate and juxtaposed units, but instead as a cooperative molecular network.

## 2. Purines during Embryogenesis and Development

In recent years, there has been increasing evidence showing that purines play an important role in controlling embryo development and organogenesis. Changes in the expression of enzymes that control the metabolism of purines (such as ectonucleotidase and adenosine deaminase) and/or receptors that encode the message are a crucial step for the control of different factors of signal transduction, which in turn ensure the proper embryo development [11]. P1, P2Y and P2X receptor subtypes are involved in the development of different organs i.e., heart, blood vessels, skeletal muscle, brain and so on [12]. In the growth and maturation of excitable cells, particularly in neuronal genesis, the release of intracellular Ca^2+^ is induced by mesoderm cells through the intervention of specific P2Y purinergic receptors probably mediated by ATP [13].

Purinergic control of neural development is not limited to early phases of prenatal life, but is also maintained later when it plays a fundamental role in controlling the maturation of glial components such as oligodendrocytes [14]. When brain development is complete, some purinergic mechanisms could be silenced, but could be re-activated in adult brain after injury, suggesting a role for purines in regeneration and self-repair [15,16].

In addition, stem cells are able to originate mature neurons and sequentially express the different types of purinergic receptors. Activation of the correct sequence of these receptors is directly related not only to differentiation into neurons or glial cells, but also to their positioning in the developing brain [17].

In the early stages of the embryonic development of skeletal muscle, the maturation of the nicotinic receptors for acetylcholine is mainly regulated by autocrine and paracrine processes of the purinergic type, which control the maturation of the muscle tissue [18]. In this phase, the expression of P2X receptors coincides with the formation of neuromuscular junctions. During this stage, ATP acts as a postsynaptic facilitator of the neuromuscular junction through the activation of P2X receptors. In contrast, in the mature junction, ATP acts as a modulator at both presynaptic and postsynaptic levels. It has also been shown that ACh and ATP are both released from the presynaptic portion through exocytosis. Later, adenosine produced extracellularly by a deamination mechanism is recaptured at presynaptic level for reconstitution of the ATP storage [19]. In the mature fibers, Adenosine, which is formed extracellularly and recaptured in presinaptic portion via A1 receptors, inhibits the further release of Ach [20]. In addition, it has been shown that at the presynaptic level, there are both A1 receptors with inhibitory effect and A2A receptors that facilitate the adenosine action. A1 receptors are more abundant in adults, while A2A ones are more present in newborns [21].

## 3. Mechanisms for the Extracellular Pool Formation

In addition to synaptic transmission, intracellular nucleotides can be released by non-specific mechanisms in response to various conditions of cellular stress due to alterations of cell membrane permeability. The nucleotides released from damaged cells act as chemotaxis signals that guide the migration of phagocytes to the injury sites leading to the removal of necrotic debris [1]. On the other hand, the increase in the extracellular pool of nucleotides and nucleosides may also be due to the existence of specific mechanisms of transport through the cell membrane essentially consisting of processes of vesicular exocytosis, opening of specific channels and the activation of transporters [22].

In particular, ATP produced in the cell can be released through microvesicles derived from plasma membranes, connexin or pannexin channels, specific ATP binding cassette carriers and calcium channels. ATP is trapped in the vesicles during vesicle formation. Then, exocytosis occurs through the canonical complex Ca^2+^-activated receptor factor named SNARE. In addition, an ATP synthase plasma membrane has also been suggested to contribute to the increase of ATP in the extracellular space [23]. Some years ago, it was shown that, in addition to intracellular regulation of G proteins, GBPs have important extracellular effects in different tissues, mainly in the nervous system [24]. Indeed, it was found that GTP was stored in neurons, even in synaptic vesicles, suggesting for it a role as a neurotransmitter similar to that described for ATP in neuromuscular synapses. GBPs would also modulate glutamate-induced cell responses both in physiological and pathological conditions. Furthermore, in addition to a now accepted role of modulation in the nervous system and in excitable tissues in general, it has been shown that ATP [25] and extracellular GTP [26] also play a role in renal physiology. These results allow us to suppose, as was done for ATP, the existence of a purinergic system based on guanine [27].

## 4. Specific Effects of GBPs in Nervous Tissue

GBPs exert several extracellular trophic effects on neural and/or glial cells not related to G proteins and having a multiple consequences on development and reparative mechanisms of nervous system [1]. Thus, nowadays it is possible to propose a specific guanine-based purinergic system in addition to the well-characterized adenine-based purinergic system [28]. In particular, Guanosine has evident neuroprotective properties; it is physiologically released in the brain as the product of the extracellular metabolism of GTP and increases its concentration during pathological events such as ischemic stroke, Alzheimer’s disease and Parkinson’s disease. In these conditions, the Guanosine (but also other GTP-related nucleotides) acts by reducing neuroinflammation, oxidative stress and excitotoxicity [29].

Current evidence suggests that guanine-based purines modulate glutamatergic parameters, such as glutamate uptake by astrocytes and synaptic vesicles, seizures induced by glutamatergic agents and in response to ischemia and excitotoxicity [30]. Glutamatergic neurotransmission is present in most mammalian excitatory synapses and plays a key role in the central nervous system. When secreted in excess, it can induce excitotoxicity, which is present in several neuropathology. Guanosine acts precisely on this system by inhibiting the secretion of the vesicles containing the neurotransmitter, thus inducing a protective effect not only in mammals but also in other species such as *C. elegans* [31].

In addition, other studies showed that guanosine would also modulate glutamate transporter activity and that guanine nucleotides are able to displace glutamate from its receptors [32]. As postulated by Tasca et al. (2018), “the identification of the extracellular actions of GBPs as intercellular messengers was possible due to five pieces of evidences: (1) GBPs can be found in the extracellular space, where they are released upon certain harmful conditions; (2) hydrolization of extracellular guanine nucleotides leads to the formation of guanosine and guanine; (3) GTP is stored in synaptic vesicles; (4) glutamate binding is displaced by guanine nucleotides; and (5) GBPs modulate glutamate transporter activity. Based on these findings, it can be concluded that GBPs act as endogenous modulators of glutamatergic transmission, and it might result in the potentially critical role that these molecules play in neuroprotection.” [1] (p. 3).

Additionally, guanine-based purines have important trophic functions affecting the development, structure and vitality of neural cells. These effects are mainly due to Guanosine present in the extracellular space, originating from the catabolism of the nucleotides of guanine by the action of ectonucleotidase. Thus, the thesis that GBPs are neuromodulators has taken on the appearance of a well-established theory supported by much experimental evidence obtained from both in vitro and in vivo experiments [33]. A particularly interesting model for the study of the GBPs-induced neuromodulation, is the use of samples derived from aged subjects. Souza and collaborators have shown that guanosine, through interactions with the glutamatergic system, exerts a significant protective effect in cell cultures from Wistar rats of different age groups [34].

The first experiments that demonstrated a modulatory effect of GBPs were carried out in the early 1990s both in primary cultures of mouse astrocytes and in human continuous cell lines with neuronal characteristics [35]. These authors showed that the presence in the extracellular space of GBPs induced an increase of NGF synthesis and release from *neopallium* astrocytes [36], and significantly stimulated the neurite outgrowth induced by nerve growth factor (NGF) in PC12 cells. Interestingly, this effect was mediated by an increase in intracellular Ca²⁺ levels and membrane hyperpolarization [37]. As consequence of the GTP-dependent Ca^2+^ increase, an early activation of extracellular-regulated kinases and phosphoinositide 3-kinase was also activated [38]. Together, these results suggest that GTP-induced differentiation is strictly dependent on a cooperative mechanism in the signal transduction between NGF and GTP after the purine binding on specific sites [39]. Similarly, guanosine also induced neurite outgrowth in cerebellar neurons, under hypoxic conditions [40]. Guanosine and GMP were also able to promote the reorganization of extracellular matrix proteins in astrocytes and increase the number of neurons in a neuron-astrocyte co-culture system through the involvement of A2A adenosine receptors and extracellular signaling pathways formed by several kinases as extracellular-regulated kinases, calcium-calmodulin-dependent kinase, protein kinase C and protein kinase A [41]. Finally, in SH-SY5Y neuroblastoma cells, a model for studying neuronal differentiation, 0.3 mM Guanosine or GTP induced a significant increase in the number of cells bearing neurites and increased neurite length. Western blot analyses confirmed that purines induced differentiation; cells exposed to purines showed increases in the levels of GAP43, MAP2 and tyrosine hydroxylase. Cytofluorimetric analyses also indicated an anti-proliferative effect of purines, and a concentration-dependent accumulation of cells in S-phase [42].

## 5. The Effects of GBPs in Muscle Cells

From what we have described so far, it seems evident that Guanosine (but also guanine) is in the category of GBPs, which is a category for derivatives that have a more significant effect on the protection and/or repair of nervous cells, while GTP and other phosphorylated derivatives seem to play a significant but marginal role only in neuro-modulation.

In contrast, the presence of extracellular Guanosine triphosphate (but also of other mono and di-phosphate nucleotides) in muscle cells takes on a preponderant role while the non-phosphorylated bases seem even ineffective. In fact, in rat hypoxia-conditioned cardio-myocytes the presence in the growth medium of different nucleotides (GTP; GDP; uridine triphosphate, UTP; thymidine triphosphate, TTP, etc.) induced cardio-protective effects by preventing free radicals formation and preserving mitochondrial activity. It is important to note that the pharmacological blockage of P2 receptors or the use of receptor knockouts cells did not abolish this effect which was completely lacking if, instead of phosphorylated nucleotides, Guanosine was present [43].

Several years ago, it was also shown that the presence of GBPs in the extracellular milieu induced positive changes in contractile capacity in frog fibers. The results of these experiments showed that several guanosine nucleotides can increase the develop of twitch tension, and that this increase was independent of external Ca^2+^ concentration [44].

Data that are even more recently obtained in unicellular ciliates, such as for *Paramecium* and *Tetrahymena*, seem to agree with previous studies, indicating a positive action on the contractile capacity of cells in the presence of extracellular GBPs. In these organisms, both ATP and GTP induced depolarization waves starting from the formation of Ca^2+^-dependent receptor potentials that are the basis of jerking movements. It is noteworthy that both ciliates show the presence of high affinity binding sites for both nucleotides, but GTP does not compete for ATP binding and vice versa [45].

A series of experiments conducted in mouse cell lines able to differentiate according to the skeletal muscle phenotype (C2C12), have allowed us to characterize the effects that the presence of GBPs, especially GTP, have on the differentiation processes that lead the undifferentiated cells (myoblasts) to merge to build myotubes. In this substrate, GTP recognizes two specific binding sites on the surface of myoblasts membranes: a high affinity site (Kd = 15.4 ± 4.6 μM); and a low affinity site (Kd = 170 ± 94.5 μM). By contrast, in myotube membranes, only one low affinity binding site for GTP (Kd = 169 ± 39 μM) is present. In both two sites detected in myoblasts, GTP was not displaced by ATP or UTP, but it was affected by treatments with suramin or Reactive Blue 2, two non-selective purine receptor antagonists [46]. As a result of the GTP binding, a metabotropic cascade is activated, which leads to a transient increase in intracellular Ca^2+^ due to an increase of intermediate Ca^2+^-activated K^+^ currents. This event is strictly related to the synthesis of the myosin heavy chain and then to the differentiation processes [47]. More recently, it has been shown that in the early stages of differentiation, GTP regulates many genes specifically involved in myogenesis including *Pp3ca*, *Gsk3b* and *Pax7*. This indicates a possible function for the nucleotide as a differentiation regulator in these cells [48].

**Figure 2:**
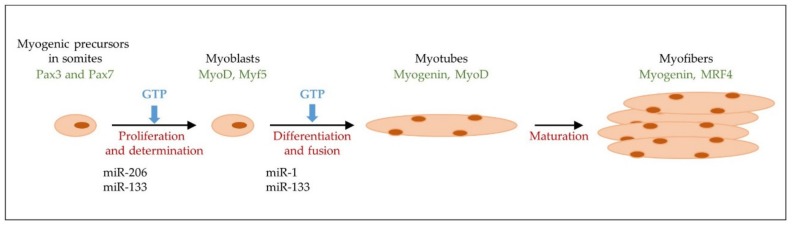
A schematic representation of myogenesis. The phases of myogenesis, main transcription factors, miRNAs and GTP involved in the development of the mature muscle fiber are shown.

There is also evidence that the presence of extracellular GTP is capable of inducing myogenic differentiation, even in adult stem cells (satellite cells) derived from human muscle. In these substrates, incubation in the presence of GTP upregulates some miRNAs such as miR133a and miR133b, known as myogenic factors. It also induces the release of exosomes containing guanosine-based purines in the same human myogenic cells (Figure 2) [49].

## 6. Concluding Remarks

It has become increasingly evident that among purine nucleotides, nucleotides based on guanine, primarily GTP, act as independent inductors and/or regulators in excitable tissues.

This is not only during development but also after organogenesis completion and during the adult phase.

In addition, GBPs are able to directly regulate many stages of regenerative and differentiating processes, even in human staminal cells. For this reason, it is possible to hypothesize for GTP and its metabolites a key role also in processes that lead, during maturity, to the preservation or restoration of altered functions due to physiological events such as senescence or pathological ones such as degenerative processes.

An important part of the studies concerning the biological processes regulated by purines is addressed to the knowledge of the mechanisms that lead to the recognition of membrane receptors.

As far as adenine purines are concerned, these studies have allowed identifying at least 15 different subtypes involving different transductive processes. In particular, two seem to be better defined: (1) those based on the interaction with specific channels and (2) the most complex systems based on the presence of G-proteins and mediated by the increase of intracellular Ca^2+^.

With regard to GBPs, no exclusive receptors have yet been identified for these purines, although research being carried out with innovative techniques in various laboratories gives hope for the near future. To date, however, no specific receptor for GTP or its metabolites has been cloned, even if specific binding sites with distinct characteristics of GTP affinity were detected both in muscle and in neural cell lines.

Even the mechanisms of cross-regulation between the two different purines (ABPs and GBPs) are still largely unknown and the cellular regulation influenced by purines is incomplete, more recently, Dal Cin et al. showed that in astrocyte, guanosine avoids oxidative damage during ischemic events via adenosine A_1_ and A2A receptors and regulation of survival signaling pathways [32]. It now seems possible to prospect the existence of specific signaling pathways for nucleotides based on guanosine, which is able to modulate the intensity and duration of the intracellular signal. As a result, the current state of international research in this field leads to substantial optimism. In our opinion, it will be possible, in the near future, to have clearer answers on the role that GTP plays in the functional capacity of excitable tissues.

This could also open up very interesting perspectives for the definition of many pathological states, in particular the degenerative ones, that currently present interpretative lacunae which are not easy to overcome with current knowledge.

## Abbreviations

ABP	Adenine-based purines
GBP	Guanosine-based purines
ATP	Adenosine triphosphate
ADP	Adenosine diphosphate
AMP	Adenosine monophosphate
GTP	Guanosine triphosphate
GDP	Guanosine diphosphate
GMP	Guanosine monophosphate
IMP	Inosine monophosphate
ACh	Acetylcholine
NGF	Nerve growth factor
UTP	Uridine triphosphate
TTP	Thymidine triphosphate
